# Total, Neutral, and Polar Lipids of Brewing Ingredients, By-Products and Beer: Evaluation of Antithrombotic Activities

**DOI:** 10.3390/foods8050171

**Published:** 2019-05-20

**Authors:** Ronan Lordan, Eoin O’Keeffe, Alexandros Tsoupras, Ioannis Zabetakis

**Affiliations:** Department of Biological Sciences, University of Limerick, Limerick V94 T9PX, Ireland; eoin.okeeffe@ul.ie (E.O.); Alexandros.Tsoupras@ul.ie (A.T.); Ioannis.Zabetakis@ul.ie (I.Z.)

**Keywords:** fermentation, beer, brewer’s spent grain, hops, polar lipids, platelet-activating factor, thrombin, cardiovascular disease, antithrombotic

## Abstract

The in vitro antithrombotic properties of polar lipid constituents of malted grain (MG), pelleted hops (PH), brewer’s spent grain (BSG), spent hops (SH), wort, and bottled beer from the same production line were assessed in human platelets. The total lipids (TL) were extracted according to the Bligh and Dyer method and further separated into the total neutral lipids (TNL) and total polar lipids (TPL) extracts by counter-current distribution. The TL, TNL, and TPL extracts of all samples were assessed for their ability to inhibit platelet-activating factor (PAF) and thrombin-induced human platelet aggregation. The raw materials, by-products, wort, and beer lipid extracts all exhibited antithrombotic properties against PAF and thrombin. However, the beer TPL exhibited the lowest IC_50_ values against PAF-induced (7.8 ± 3.9 µg) and thrombin-induced (4.3 ± 3.0 µg) platelet aggregation indicating that these polar lipids were the most antithrombotic. The lipid extracts tended to be more bioactive against the thrombin pathway. The fatty acid content of all the TPL extracts were assessed using GC-MS. The fatty acid composition of the most bioactive TPL extracts, the wort and the beer, shared similar fatty acid profiles. Indeed, it was noted that fermentation seems to play a role in increasing the antithrombotic properties of polar lipids against PAF and thrombin by moderately altering the polar lipid fatty acid composition. Furthermore, the use of brewing by-products as a source of functional cardioprotective lipids warrants further investigation and valorisation.

## 1. Introduction

Cardiovascular diseases (CVD) are the leading cause of mortality globally, where diet and lifestyle are key modifiable risk factors [1]. The harmful effects of alcohol consumption have been well established, as excessive alcohol consumption has been linked to several chronic diseases, including cancer [2,3]. However, moderate alcohol consumption (up to 16 g alcohol/day) has been associated with cardiovascular health benefits, including reduced fasting glucose and blood insulin sensitivity versus non-alcoholic beer in healthy men [4]. Indeed, consumption of 1–2 alcoholic beverages/day is associated with reduced fibrinogen levels, reduced platelet aggregation, and an increase in high-density lipoprotein (HDL). These effects have mostly been attributed to the ethanol content and the presence of phenolic compounds [5,6,7]. Epidemiological studies demonstrate that moderate alcohol consumption reduced cardiovascular risk factors, morbidity, and mortality following a dose-effect relationship that is characterised by a J-shaped curve [6].

Atherosclerosis is the first step in the development of CVD [1]. Platelet-activating factor (PAF) is a potent platelet agonist and inflammatory mediator implicated in the onset and progression of atherosclerosis [8,9]. PAF and PAF-like molecules carry out their functions by binding to the PAF-receptor (PAF-R), which is expressed in various cell types, including platelets, endothelial cells, neutrophils, and macrophages [1]. Activation of the PAF-R leads to the induction of multiple inflammatory pathways and platelet activation [1].

Thrombin is another important mediator of platelet activation [10]. Thrombin is a serine protease that participates in the coagulation cascade, activating factors V, VIII, XI, and XII, converting fibrinogen to fibrin, and activating other cell types [10,11]. Both PAF and thrombin are produced during coagulation and inflammation and play a crucial role in platelet activation and thrombus formation via G protein-coupled receptors [12]. Previously these two pathways were considered independent. However, recent evidence suggests that there is a crosstalk between coagulatory and inflammatory pathways during pathological processes, whereby inflammation leads to platelet activation in a reciprocal fashion [13]. Considering the important roles that PAF and thrombin hold at the nexus of coagulation and inflammation, antiplatelet and anti-inflammatory therapeutic and preventative strategies are required to prevent the development of chronic diseases such as CVD. Within this concept, several compounds of natural origin [1] can inhibit the binding of PAF to the PAF-R, which ameliorates the PAF inflammatory and prothrombotic response. Notably, polar lipid constituents of ale, lager, and stout have exhibited potent anti-PAF and antithrombotic properties [14]. However, little is known about the origin of these beer-derived antithrombotic polar lipid microconstituents and their effects against the thrombin pathway.

Beer is the most consumed alcoholic beverage globally. The raw materials used in beer production influence the different characteristics and properties of the beer [4]. Beers are produced from malted barley, water, hops (*Humulus lupulus*), and yeast (*Saccharomyces cerevisiae*). Barley (*Hordeum vulgare*) contains 2–4% (dry weight) lipid depending on various factors [15]. Commercial malts can contain up to 3.4% lipid. Approximately 70–90% of the fatty acid content of the barley and malt is triglycerides, 10–20% are sterol compounds, and approximately 10% are free fatty acids. The free lipid composition of the barley grain is approximately 68–75% neutral lipids, 7–26% glycolipids, and 9–18% phospholipids depending on the cultivar [16]. Germination of the barley and the mashing process can lead to the loss of lipid due to the release of fatty acids via the hydrolysis of triglycerides, which are then metabolised. The resulting fatty acids, mono-, and diglycerides do not tend to accumulate in the malt, and thus are not found in significant quantities in the finished product [17]. Moreover, several phospholipids can form complexes with amylose in starch before the brewing process [18]. A significant proportion of the lipid content is lost to the spent grains generated during the brewing process, and so the wort and beer contains low levels of lipid [19]. There is only a trace amount of lipid that remains in the final beer product, which are generally considered undesirable due to their impact on the formation of haze, the stability of beer foam, and the development of unfavourable flavours during conditioning [17,20].

Brewing yeasts also have the capacity to synthesise and alter several lipid species, including phospholipids and polyunsaturated fatty acids (PUFA) [21], which play a crucial role in the metabolic pathways and regulation of lipid catabolism and anabolism in yeast [17]. Furthermore, there is an increased concentration of stearic, *cis*-oleic, and linolenic acid in the wort as a result of endogenous lipase activity that releases free fatty acids from triglycerides and phospholipids in the mashing and malting process [21,22]. In addition, the composition of the malt and lauter turbidity can lead to the formation of triglycerides, diglycerides, monoglycerides, free fatty acids, phospholipids, and sphingolipids during the fermentation process [23].

The unfortunate consequence of beer production is the generation of brewing wastes and by-products, which are a significant environmental challenge, but may be valorised for the development of novel products [24]. Industrial-scale food processing by-products are increasingly being viewed as potential sources of bioactive ingredients. One such example in the brewing industry by-product previously sent to landfill is BSG [25], which is now primarily used for animal feed. The lipid content of BSG is approximately 10.0–13.5% (*w/w* of BSG samples) [26], where it is estimated that 9.1% of the total lipids are phospholipids [27].

Considering little is known about the fatty acid composition of bioactive polar lipid microconstituents of beer or brewing by-products, the aim of this study was to assess the antithrombotic properties and fatty acid composition of lipid extracts sampled at various stages of the brewing process, from the brewing raw materials (malted grain and hops), the by-products (BSG and spent hops), the wort, and the finished beer product from a commercial beer produced in Ireland.

## 2. Materials and Methods

### 2.1. Chemicals and Reagents

All glassware, chemicals, and solvents were of analytical grade and purchased from Fisher Scientific Ireland Ltd. (Dublin, Ireland). All reagents used for platelet aggregation, including bovine serum albumin (BSA), standard PAF, and standard thrombin, were high purity and purchased from Sigma-Aldrich (Wicklow, Ireland). Blood consumables, including needles (20G) and 8.2 mL sodium citrate S-monovettes, were purchased from Sarstedt Ltd. (Wexford, Ireland). For GC-MS, a pre-derivatised Supelco™ 37-component FAME standard mix, pre-derivatised heptadecanoic acid, and pre-derivatised heneicosanoic acid was purchased from Sigma-Aldrich (Wicklow, Ireland). All platelet aggregometry consumables were purchased from Labmedics LLP (Abingdon on Thames, UK). All GC-MS consumables were purchased from Apex Scientific Ltd. (Kildare, Ireland).

### 2.2. Beer Production and Samples Assessed

The beer, wort, raw materials, and brewing by-products used for this study were obtained from the Munster Brewery facility (Youghal, Co. Cork, Ireland). The samples assessed in this study were pelleted hops (PH), spent hops (SH), malted grains (MG), brewer’s spent grains (BSG), wort, and the beer itself. All the raw materials, wort, and by-products obtained for this study correspond to the same batch and production line of the beer that was produced for and tested in this study. The beer is an organically produced Irish red ale for commercial sale under the name ‘12 Towers’ brewed in accordance with organic standards certified by the Irish Organic Association.

The beer production is described in brief as follows. Of the malts used, approximately 90% of the overall malt was organic pale ale malt (Maris Otter malt) was kiln dried at 90–95 °C to produce the base malt of the beer with a maximum moisture content of 4.5% and a European Brewing Convention (EBC) colour scale between 7 and 10 (Castle Malting Ltd., Beloeil, Belgium). A small amount of organic roasted barley (approximately 10% of the total malt used), which was kiln dried to 230 °C that produced a roasted grain that had a maximum moisture content of 4.5% and was a colour rating between 1000–1400 EBC. Once milled by the brewery, the grains were mashed in the mash tun. To formulate the wort, 4 L of water was added per kg of crushed grain, which was steeped in the mash tun at 66 °C for 60 min. This process activated the α- and β-amylase enzymes (among others) that convert the starch to simple sugars to produce the so-called sweet wort. At the end of the mash, the lautering process begins by raising the temperature to 77–79 °C to denature the enzymes by adding heated sparge water, which also weakens the gravity of the wort runoff. This process takes approximately 3–4 h. Once Lautering was complete a sample of the BSG was stored. A brewer’s hydrometer was used to measure the original gravity (OG) of the first running, which can be as high as 1.080 and also subsequently to confirm that the kettle wort is at an acceptable gravity prior to commencing boiling. The OG prior to fermentation was 1.042 and final gravity (FG) after fermentation was 1.010. These hydrometer readings are used to measure the fermentable and unfermentable substances the in wort before and during fermentation and to calculate the beers final alcoholic content (alcohol by volume or ABV) when OG is compared to the FG. Runnings from the mash tun were transferred to the kettle and once complete, the wort was heated to 100 °C and brought to the boil for 1 h, which sterilised the wort, denatured any remaining enzymes, and allowed for caramelisation of the sugars for flavour enhancement.

At the start of the boil a specific amount of organic pelleted hops (PH: Organic Goldings Hops, Charles Faram Ltd., Worcester, UK) was calculated based on flavour rating (approximately 200 g/hL) of the hops for each batch and was added to the kettle, which was boiled for a further hour. These pelleted hops are strobiles from the female hop plant that are air-dried and pelleted, and thus extremely concentrated in comparison to fresh strobiles. Thereafter, the wort underwent whirlpooling a rapid cooling process by being passed through a heat exchanger, which reduced the temperature of the wort to 20 °C. A sample of the wort was taken at this point. After whirlpooling when the wort had been moved on in the process, a sample of the SH was taken. The SH sample in reality is not purely hops, it is also known as the trub, which consists of primarily hop debris (10–20%), but is also a source of sedimented protein (50–70%), phenolic compounds (5–10%), carbohydrates (4–8%), and fatty acids (1–2%) originating from the hops and residues from the barley processing [28]. The cooled wort was transferred to the primary fermenter and was held at 20 °C. At this temperature, the yeast, a dehydrated *Saccharomyces cerevisiae* (Nottingham High Performance Ale Yeast, Lallemand Inc., Burton upon Trent, UK) was diluted in sterilised water and pitched (80–100 g per hL) into the filled fermentation tank. This is a top fermenting yeast that is commonly used to produce a wide variety of beers, including ambers, porters, stouts, and pale ales. The fermentation ran for four days. Following fermentation, a conditioning period was allowed to take place at 12 °C for approximately 7 days. After conditioning, the beer underwent cold crashing. In this process, the temperature of the conditioned beer is reduced to between 0–2 °C over 3–4 days. The process of cold crashing promotes the flocculation of yeast, which sink to the bottom of the tank due to gravity and are removed, thus increasing the clarity of the beer naturally without the need for additives. Indeed, cold crashing is also desirable due to its effects on other suspended particles, such as tannins, polyphenols, and proteins that can also flocculate and settle at the bottom of the tanks, preventing the undesirable phenomenon of chill haze. Once cold crashing was complete, the beer was bottled with a 2 g of 100% fermentable organic dextrose (Charles Faram Ltd., Worcester, UK) added to each bottle to encourage carbonation by any remaining yeast in the beer. The carbonation process takes approximately 14 days at room temperature until an optimal level of 2.2–2.5 volumes of CO_2_ was achieved. Once complete the bottles were placed in cold storage (0–4 °C) for a short period of time until ready to distribute.

**Figure 1:**
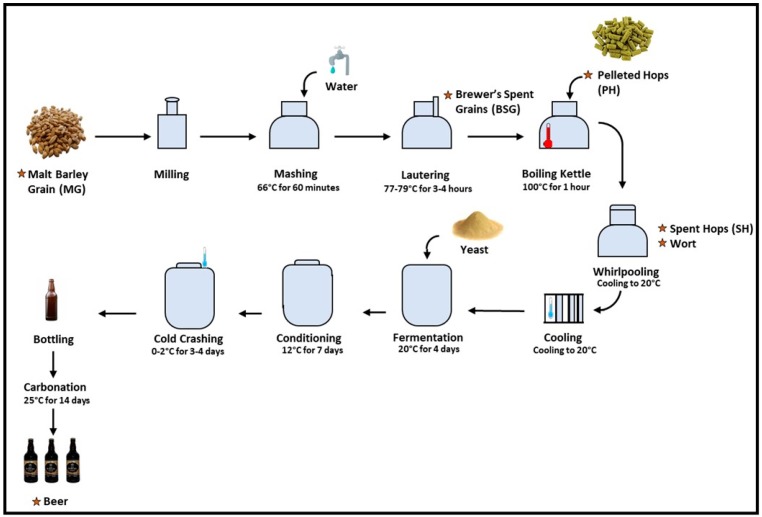
A schematic of the brewing process for the production of Irish red ale outlining the sampling points for the brewing materials, by-products, wort, and beer as highlighted with a star.

The finished bottled beer product, the wort, and the various by-products collected were placed in airtight containers and transported to the laboratory. The beer and wort were extracted on the day of arrival to the laboratory and the other by-products and raw materials were stored at −20 °C for a maximum of three weeks until required for extraction and analysis. All samples were taken in triplicate from different batches of the beer production process. The brewing process and sampling points are outlined in Figure 1.

### 2.3. Extraction and Isolation of the TL, TNL, and TPL Extracts

The total lipids (TL) of all samples were extracted in triplicate according to the Bligh and Dyer [29] method. Notably, the lipids from the brewing materials, by-products, wort, and beer were extracted from different batches of production. A tenth of each TL was stored under nitrogen at −20 °C and the remaining lipid was subjected to counter-current distribution as previously described [14] to obtain the total polar lipid (TPL) and the total neutral lipid (TNL) extracts. All extracts were stored under nitrogen at −20 °C until required for a maximum of 6 months as previously described [14].

### 2.4. Platelet Aggregation Assay

The in vitro assessment of PAF and thrombin-induced platelet aggregation was carried out as previously described [14,30]. In brief, healthy human volunteers (*N* = 12) free from any form of antiplatelet therapy gave informed written consent and all protocols were executed in accordance with the Declaration of Helsinki following ethical approval by the University of Limerick Ethics Committee. Participants provided 50 mL or blood following an overnight fast (>8 h). The blood was drawn via venepuncture of the median cubital vein using a 20G needle into evacuated sodium citrate S-monovettes via the aspiration method (0.106 mol/L in a 1:10 ratio of citrate to blood). To obtain the platelet-rich plasma, S-monovettes were immediately centrifuged at 180× *g* for 18 min at 24 °C (Eppendorf 5702 R, Eppendorf Ltd, Stevenage, UK). A second centrifugation at 1500× *g* for 20 min at 24 °C was carried out to obtain the platelet-poor plasma (PPP). The PRP was standardised to 500,000 platelets µL^−1^ using a Shimadzu UV-1800 spectrophotometer (Kyoto, Japan), prior to analysis on a Chronolog-490 two channel platelet aggregometer (Chronolog, Havertown, PA, USA), coupled to the specialised AGGRO/LINK software package. Prior to testing, lipid samples and standard PAF were dissolved in a solution of BSA-saline (2.5 mg BSA/mL saline), while aliquots of stock solutions of active thrombin were diluted in appropriate amounts of physiological saline to obtain solutions of active thrombin with a final concentration in the aggregometer cuvette, ranging from 0.01 mU/mL NIH (National Institute of Health). The final concentration of PAF in the cuvette ranges from 1–5 × 10^−8^ M. Then, 250 µL of PRP was added to an aggregometer cuvette at 37 °C with stirring at 1000 rpm. The PRP was calibrated using the PPP as a blank. PAF and thrombin were added to the cuvettes in order to induce maximum reversible aggregation in the absence of any lipid samples. For each lipid sample, the mass of lipid required to inhibit 50% the PAF or thrombin-induced aggregation was calculated. Subsequently, the IC_50_ was calculated as previously described [14,30]. Platelet aggregation experiments for each TPL, TNL, and TL extract was carried out in triplicate against both PAF and thrombin as previously described [30,31].

### 2.5. Gas Chromatography-Mass Spectrometry

The preparation and analysis of the fatty acid methyl esters (FAME) were carried out in triplicate using 35 mg of the TPL samples of all samples as previously described [14]. Briefly, FAMEs were derivatised using a 0.5 M KOH CH_3_OH 90% solution and extracted with n-hexane. The analysis was carried out using the internal standard method (Heneicosanoic acid—21:0) as previously described for other beverage analysis [14]. A five-point calibration curve was prepared using heneicosanoic acid (21:0 500 ppm injections) and five solutions of heptadecanoic acid (17:0—50, 100, 200, 400, and 800 ppm) methyl ester standards. Five 1 µL injections of each solution were analysed using a Varian 410-GC coupled to a Varian 210-MS equipped with a split/splitless injector (Agilent Technologies, Santa Clara, CA, USA). Separation of the FAME was conducted on an Agilent J&W DB-23 fused silica capillary column (60 m, ‘0.25 mm i.d.’ 0.25 µm f.t.; Agilent Technologies). The ratio of the mean 17:0 to that of the internal standard (21:0) was used as the y-axis variable, while the concentration (ppm) of 17:0 was used as the x-axis variable of the calibration curve. The equation describing the curve was: y = 0.0041x + 0.12, with a *R*^2^ = 0.9969, where the ratio of the area of the analyte peak to that of the internal standard represents the y value for the equation of the calibration curve and the x value represents the analyte concentration of a selected fatty acid in the lipid sample. The injector temperature of the Varian 410-GC and 210-MS was set at 230 °C with a split ratio of 1:20. The carrier gas was high purity helium with a liner flow rate of 1 mL/min. The oven temperature was initially programmed to 100 °C for 5 min, raised to 240 °C at 3 °C/min, and finally held isothermal at 240 °C for 10 min. Identification of FAME was achieved using a pre-derivatised standard 37-component FAME sample mix and comparison of the retention times and mass spectra of relative peaks with the aid of the Varian Star Chromatography Workstation Version 6 software (Agilent Technologies) and a NIST library of mass spectra (Gaithersburg, MD, USA). The percentage of each fatty acid was calculated using the peak area of the samples corrected by the respective response factors. Analyses were carried out in triplicate.

### 2.6. Statistical Analysis

All biological experimental analyses, extractions, and GC-MS analyses for each lipid sample were sample were completed in triplicate. The obtained results were expressed as a mean value ± standard deviation (SD). One-way analysis of variance (ANOVA) and the Tukey’s honest significant difference (HSD) multiple comparison post-hoc test was used to determine the significant statistical differences between the analyses (SPSS Inc., Chicago, IL, USA).

## 3. Results

### 3.1. Lipid Extraction and Fractionation of Beer, and Brewing Materials

The TL, TNL, and TPL content of the beer and brewing materials are shown in Table 1 expressed in either mg/100g or g/100 g of TL, TNL, and TPL. The TNL and TPL are also expressed as a percentage of the TL. The MG and BSG were both considerably low in TL, but the TPL accounted for a substantial amount of the MG, of which just over half seems to be lost to the Brewer’s spent grain in the brewing process. The largest amount of TL and TPL present in g/100 g of all samples was the PH, which was significantly higher than all the other samples. Notably the SH had a considerably lower quantity of TL and TPL than the PH, indicating that these lipids may have been extracted and diluted into the wort. However, both the wort and the beer contained extremely low amounts of lipid, the majority of which were polar lipids, results that are in accordance with previously published research [14,32,33].

### 3.2. Gas Chromatography-Mass Spectrometry Analysis

While the general lipid composition of beer, wort, barley, and malts have been comprehensively characterised by Bravi and colleagues [32,33,34], to the best of our knowledge the fatty acid profile of the polar lipids specifically have not been previously reported. Therefore, the fatty acid profile of each TPL extract were assessed by GC-MS (Table 2). Beer consists of many volatile and non-volatile compounds that affect the beer flavour and bioactivities [14,32]. Many of the volatiles and fatty acids in beer are synthesised by yeasts during fermentation, whereas others are derived from the raw materials [32]. Research demonstrates that the dominant fatty acids in the TL of barley grains, wort, and beer are palmitic, stearic, *cis*-oleic, linoleic, and α-linolenic acids [35]. However, little is known about the fatty acid profile of the polar lipid fractions of the raw materials, wort, or the beer itself.

In this study, the MG and BSG had similar TPL fatty acid profiles. Notably, there were statistically significant differences between the MG and BSG in the percentage of palmitic and linoleic acids, where both were higher percentages in the BSG than the MG TPL. Furthermore eicosenoic acid and docosahexaenoic acids were present as a low percentage of the MG but were not detected in the BSG TPL. Additionally, there were significant differences in the TPL fatty acid composition between the MG, BSG, and the beer, namely the TPL fatty acids differed by a higher percentage of SFA and a lower percentage of monounsaturated fatty acids (MUFA) and polyunsaturated fatty acids (PUFA) in the beer TPL in contrast to the MG and BSG. While the MUFA and PUFA were present in lower amounts in the beer compared to the MG and BSG, arachidonic acid was present in the beer in notably high amounts (4.9%) but was not present in the MG or BSG. Furthermore, myristic and stearic acid were also present in a significantly high percentage of the beer TPL in contrast to the MG and BSG. The MG and the wort also differ in composition, as there was a higher percentage of palmitic, stearic, arachidonic, and eicosapentaenoic acids and a lower percentage of linoleic, arachidic, eicosenoic acid, and docosahexaenoic acid with no phytochemicals detected in the MG, BSG, or wort TPL.

Another contributing factor to the fatty acid composition of beer is the hops. Hops are known for their high concentrations of volatile components that can impart bitter flavours and aroma to the wort and final product in the brewing process [36]. However, there is relatively little literature addressing the fatty acid compositions of the TL or TPL of hops. In this study, there were significant differences between the fatty acid compositions of the TPL of the PH and the SH. While the total percentage of PUFA in the TPL of the PH and SH were similar, there was a significant increase in the percentage of SFA and MUFA in the SH. However, this is most likely not due to an actual rise in the levels of these fatty acids in the TPL, but may be due to the fact that the PH TPL extract contained a high amount of volatile components that seemed to significantly reduce in the SH. Considering these levels of volatile phytochemicals were not present in the wort or beer in any significant percentages, it is likely that these volatiles were lost during the boiling of the wort, which has been documented previously in studies [37]. Generally, there was a lower percentage of palmitoleic, margaric, and α-linolenic, arachidic, eicosadienoic, docosahexaenoic acids in the SH in contrast to the PH, whereas there was a significantly higher percentage of myristic, palmitic, stearic, *cis*-oleic, *trans*-oleic, and linoleic acids in the SH in contrast to the PH. Furthermore, there was a high percentage of caryophyllene and aromadendrene oxide in the PH TPL.

While the intention of this analysis was to assess the fatty acid profile of the TPL extract, volatile phytochemicals and other constituents were also detected in the TPL extracts of beer, PH, and SH in low percentages. The beer TPL extract contained the highest percentage of phytochemicals at 1.37%, which can mainly be attributed to the detection of adipic acid (or hexanedioic acid). Other compounds identified in the final beer TPL extract were β-caryophyllene, 2,4-Di-*tert*-butylphenol, and tau-muurolol in low percentages. β-caryophyllene and other essential oils were also present in the PH TPL extracts, including tau-muurolol, a cadinene sesquiterpenoid that is a plant metabolite, fungicide, and volatile oil [38], which is commonly found in hops [39]. The PH also contains β-caryophyllene, a bicyclic sesquiterpene that was also present in the beer and SH. β-caryophyllene is a characteristic essential oil of hops that is usually in high abundance in comparison to other phytochemicals [36,40] and is the most abundant phytochemical in the PH and SH TPL extract.

### 3.3. Platelet Aggregation Assay Analysis

The TL, TNL, and TPL of each extract were assessed for their ability to inhibit PAF and thrombin-induced platelet aggregation in human PRP. The results of the platelet aggregometry assay for all lipid samples are expressed as an IC_50_ value, which is the mass of the lipid sample in micrograms (µg) required to inhibit half (50%) the maximum-reversible PAF/thrombin-induced platelet aggregation. The IC_50_ results for each extract tested against PAF-induced platelet aggregation are presented in Table 3. It is clear from the data presented that overall the TL and TPL are the most bioactive fractions in the various brewing-related extracts. Overall, the TNL extracts generally exhibited poor bioactivity. Notably in the MG, the TNL was slightly more bioactive than the TL, but was less bioactive than the TPL. Furthermore, the BSG TL had an exceedingly low IC_50_, and thus a higher anti-PAF effect, in comparison to the relative effect of the TNL and TPL. It is not known why this may be the case and further research is required to ascertain whether there may be synergistic effects between the compounds extracted in the TL of the BSG. In terms of the hop extracts, TL, TNL, and TPL IC_50_ values were similar and did not show any statistically significant differences between the PH and the SH. The wort and final beer product were generally the most bioactive TL, TNL, and TPL extracts, although not statistically significantly different from each other.

The IC_50_ results for each lipid extract assessed against thrombin-induced platelet aggregation are presented in Table 4. Similarly to the results of the PAF-induced platelet aggregation assay, it is clear that overall the TL and TPL are the most bioactive fractions in the various brewing-related extracts. The IC_50_ values of the TNL of the MG, BSG, PH, and SH were high, indicating poor inhibition against thrombin. However, the TNL IC_50_ values for the wort and beer were comparable to each other and were considerably lower, and thus more effective against thrombin than the TNL extracts from all other sources. Like the TNL extracts, the TPL of the MG, BSG, PH, and SH were high, indicating poor thrombin inhibition. However, the wort and the beer exhibited extremely low IC_50_ values that were not statistically significantly different from each other. Notably, the TL extracts of the MG, BSG, PH, and SH also possess considerable inhibitory properties against thrombin, although not as potent as the beer or the wort.

## 4. Discussion

Previous research has demonstrated that commercial ale, lager, and stout possess potent anti-PAF activities as demonstrated through PAF-induced platelet aggregation assays [14]. Therefore, the aim of this study was to assess the antithrombotic activities and fatty acid composition of brewing raw materials, by-products, wort, and beer from a single production line in an active brewery.

In this study the TL, TNL, and TPL were extracted from the raw materials (MG and PH), by-products (BSG and SH), wort, and Irish red ale, all originating from the same production line. The raw materials used in beer production contain a significant amount of lipids, particularly from the malted barley and hops. However, only trace amounts remain in the final beer product [19]. The lipid content of the MG mix used as a raw material in this study was considerably low (0.7 g/100 g), where other ale malts can possess higher lipid levels between 2.8–3.4 g/100 g as reported by Anness [41]. There are several reasons why the lipid content of the MG was low in this study, including that the lipids of barley tend to form complex interactions making them tightly bound to starch [18], thus making them difficult to extract. It is possible that much of the lipid was not efficiently extracted using the Bligh and Dyer [29] method. This extraction method does not use harsh acid or heat treatments and therefore does not efficiently extract all of the lipids but was chosen for this study as an efficient method for extracting bioactive lipids against platelet aggregation. Indeed, studies have demonstrated a large variation of lipid yield between different extraction procedures, including some being more efficient than others for co-extracting non-lipid substances as part of the crude lipid content [42]. Other studies have also acknowledged that barley lipids are poorly extractable without the use of hot alcoholic extraction [43]. Therefore, further studies should consider the use of other extraction methods.

Notably, the BSG had a non-statistically significant higher lipid content than the malt, but a statistically lower TPL content (~39% of the total lipids). This is in accordance with previous research that estimates that 30% of the barley lipid content is lost during the germination of barley due to the hydrolysis of triglycerides, which are subsequently metabolised [41].

The greatest amount of lipid extracted from any sample was the PH (14.7 g/100 g) with 79% being polar compounds. In hops generally, there is a limited amount of fatty acids (1–2%) present [28]. However, the essential oil content of air-dried female hop flowers (strobiles) is generally around 0.5–3%, whereas waxes and steroids are generally present in trace to high amounts (25%) [44]. Considering that the hops used in this study were dried and pelleted, the oil and fatty acid content was highly concentrated, thus explaining the high lipid content as per Table 1. The hops were added during the boiling process to allow the essential oils present in the hops to contribute to beer flavour and aroma [40]. However, much of the essential oils present in these bittering hops are highly volatile and some are lost during the boiling process to evaporation [37], while any that remain are dispersed in the wort, but may be filtered out during clarification of the wort. At the end of the boiling process, the SH are collected within the trub. As demonstrated in Table 1, the SH contains low levels of lipids. This is most likely due to the pelleted hops being dispersed and rehydrated within the wort and the loss of some volatile compounds from the essential oils during the boiling process. Notably, there was a high percentage of caryophyllene and aromadendrene oxide in the PH TPL. Aromadendrene oxide is an oxygenated sesquiterpene that is considered an essential oil, which exhibits anticancer properties [45].

During the brewing process, a significant proportion of the lipid content is lost to the spent grains [17]. Indeed, in this study the lipid content of the beer and wort were considerably low, but the low beer TL is in accordance with previously published research [14,41]. The presence of lipids in beer is generally considered as a negative proponent due to their effect on foam stabilisation and flavour. Therefore, the reduction of lipid levels is actively reduced where possible through various parts of the clarification processes used by breweries. The low lipid content of beer generally may also be due to the fact that barley contains polar lipids and fatty acids that are closely associated with polysaccharides that create amylose-lipid complexes that are difficult to fully extract [23]. Considering the high TL and TPL content of the BSG, it is likely that this is the case and that these lipids are lost to the BSG.

The fatty acid composition of the TPL extracts of all the brewing ingredients, by-products, wort and beer was determined. While the intention of the GC-MS analysis was to assess the fatty acid profile of the TPL extract, volatile phytochemicals and other constituents were also detected in the TPL extracts of the beer, PH, and SH in low but considerable percentages. The beer TPL extract contained the highest percentage of phytochemicals at 1.37%, which can mainly be attributed to the detection of adipic acid, an unusual non-volatile, alcohol soluble, dicarboxylic acid found sparsely in nature but is used as a food additive (E355) as a firming or raising agent, which has tart flavour and is safe for human consumption in low doses [46]. As it was not an intentional additive in the production of the beer, it is not known where in the brewing process the adipic acid originates from or if it was a contaminant as it was only found in the TPL of the wort and the final beer product.

Phenolic compounds play a significant role in aroma and flavour development in beer production [47]. Present in beer, 2,4-Di-*tert*-butylphenol is a phenolic compound that is produced by a variety of plants, but can also synthesised enzymatically by *S. cerevisiae* from organic acids [48]. Interestingly it is a bioactive compound with potential anticancer effects [49], antioxidant activities, and may be preventative against the neuroinflammatory effects of amyloid beta (Aβ) in animal models of Alzheimer’s disease [50]. Considering some phenolic compounds have antiplatelet properties [51], it is yet to be determined whether 2,4-Di-*tert*-butylphenol contributed to the antithrombotic activities observed in this study. Tau-muurolol was also detected, which is a cadinene sesquiterpenoid that is a plant metabolite, fungicide, and volatile oil [52] that was detected in the beer and PH TPL extracts. β-caryophyllene, a bicyclic sesquiterpene was also present in the beer, PH, and SH. β-caryophyllene is a characteristic essential oil of hops that is usually in high abundance in comparison to other phytochemicals [36,40] and is the most abundant phytochemical in the PH and SH TPL extract. Notably, caryophyllene compounds may possess anticancer, analgesic, antioxidant, antimicrobial, and anti-inflammatory activities [53]. Indeed, caryophyllene molecules were present in abundance in essential oil extracts from 25 species of plants that demonstrated anti-platelet activity against adenosine diphosphate (ADP), arachidonic acid, and the thromboxane A_2_ agonist U46619-induced platelet aggregation in guinea pig and rat plasma [54].

PAF and thrombin-induced platelet aggregation assays were used to assess the antithrombotic activity of the TL, TNL, and TPL extracts of the brewing raw materials, by-products, wort, and beer. Generally, the TPL extracts were the most potent against PAF-induced platelet aggregation. The TL extracts exhibited considerable anti-PAF and anti-thrombin effects, but generally the TPL extracts were more potent against PAF, whereas in the case of thrombin, the TL in cases were considerably more antithrombotic than the TNL and moderately more potent that the TPL extracts. Considering, the overall poorer inhibitory effects of both the TNL and TPL extracts of the MG, BSG, PH, and SH against thrombin, the compounds present in the combined TL extract may induce synergistic effects that improve the antithrombotic properties of these extracts, as has previously been demonstrated in beer [14] and other extracts of natural origin marine extracts [55]. It can be suggested that coextracted microconstituents such as phenolic compounds and phytochemicals with potential antithrombotic activities may in part be responsible for these observations as previously demonstrated [51]. Indeed, considering the BSG possessed potent anti-PAF and anti-thrombin activities, these by-products of the brewing industry could potentially be used in the development of nutraceuticals or functional foods and animal feeds, as has previously been demonstrated using by-products of the olive oil industry [56]. Indeed, BSG contains other significant bioactive microconstituents such as peptides and phenolic compounds that exhibit antioxidant activity [26] that supports the need for further research into the valorisation of BSG as a functional product for human health.

The beer and wort extracts were the most bioactive fractions against PAF and thrombin. While not deemed statistically significantly different, the bioactivity of the TL and TPL of the wort seemed to increase considerably following fermentation. Previous studies in dairy products have shown that the fermentation process may play a role in the biosynthesis of functional antithrombotic lipids [57]. However, it has yet to be confirmed whether *S. cerevisiae* can indeed biosynthesise antithrombotic polar lipids, but this study does demonstrate that yeasts may affect the fatty acid composition of the polar lipids. Further structure activity relationship studies are required to confirm this notion.

As depicted in Table 2, there is a statistically significant increase in the percentage of polar lipids bearing fatty acids arachidonic acid and eicosapentaenoic acid (EPA) in their structures as a result or the wort fermentation. Various studies have demonstrated that polar lipids of natural origin that bear these fatty acids in their structures along with stearic, cis-oleic, and linoleic acids, all of which are present in abundance in the beer and wort TPL, exhibit potent antithrombotic properties against PAF-induced platelet aggregation [9]. Similarly, the beer (red ale) TL and TPL IC_50_ values obtained in this study were similar to those published for Smithwick’s red ale TL and TPL against PAF-induced platelet aggregation using the same methods [27]. The fatty acid compositions of the wort and beer TPL share structural resemblance to the classical PAF structure, which is generally composed of palmitic (68%), stearic (27%), or oleic (4%) acids at the *sn*-1 position, with acetic acid esterified to the *sn*-2 position, and a phosphocholine group at the *sn*-3 position [58]. Juxtaposed, the most abundant fatty acids of the TPL of the wort were palmitic (31.8%), stearic (3.6%), oleic (6.1%), and linoleic (44.8%) acids and the most abundant fatty acids present in the TPL of the beer were palmitic (32.3%), stearic (3.3%), oleic (5.6%), and linoleic (43.4%) acids. Further research is required to confirm whether there is structural homology between some polar lipids and PAF, which facilitates their binding to the PAF-R, which may account for their potent biological actions against PAF.

In contrast to the wealth of evidence demonstrating that polar lipids can inhibit the biological actions of PAF, there is little published research demonstrating the mechanisms for the antiplatelet effects of food-derived polar lipids against thrombin-induced platelet aggregation. It seems that the amphiphilic properties of these bioactive polar lipid moieties expedite their transfer from blood lipoproteins to the membranes of circulating platelets. Thus such bioactive polar lipids can either directly affect several platelet membrane receptors related to platelet activation (i.e. binding of polar lipids to the PAF-R) [31] or indirectly affect these platelet receptors. Polar lipids may indirectly affect platelet receptors through altering the microenvironment and polarisation of the phospholipid membrane, which potentially alters the affinity of a ligand to a receptor relating to platelet activation, such as thrombin [9,31,59,60]. However, further research is required to verify these potential mechanisms and to discern the structures of these compounds, in order to fully elucidate the structure activity relationships between bioactive polar lipid extracts and their overall antiplatelet effects.

## 5. Conclusions

The antithrombotic activities of lipid extracts from brewing raw materials, by-products, wort, and beer were assessed. The most bioactive anti-PAF and anti-thrombin polar lipid extracts originated from the wort and the final beer product. While not statistically significantly different, it is apparent that fermentation of the wort may play a key role in increasing the anti-PAF bioactivity of polar lipids extracted from beer. These findings are in accordance with previous studies demonstrating that fermentation plays a key role in altering the bioactivity of anti-PAF polar lipids during milk fermentation. Indeed, this research supports and furthers previously published research demonstrating the presence of potent anti-PAF polar lipids in red ale. It was also observed that some phytochemicals and phenolic compounds may contribute to antithrombotic properties of these lipid extracts. Furthermore, this is the first study to demonstrate the anti-thrombin activities of beer polar lipids, but further research is required to discern the exact structures and mechanisms responsible for these observations. Moreover, it was determined that the BSG may be a suitable brewing industry by-product for valorisation as potential nutraceuticals or functional foods for improved human cardiovascular health.

## Figures and Tables

**Table 1:** The total lipid (TL) and the total polar lipids (TPL) content of beer and brewing by-product are expressed as g/100 g and the total neutral lipid content (TNL) is expressed as mg/100 g. The TPL and TNL are also expressed as a percentage of the TL (mean ± SD, *n* = 3).

Sample	TL (g/100 g)	TNL (mg/100 g)	TNL (% TL)	TPL (g/100 g)	TPL (% TL)
MG	0.70 ± 0.10 ^a^	100 ± 30 ^a^	13.6 ± 2.9 ^c^	0.52 ± 0.05 ^a^	74.5 ± 3.5 ^b^
BSG	1.05 ± 0.19 ^a^	550 ± 110 ^b^	52.6 ± 2.5 ^e^	0.41 ± 0.09 ^a^	38.9 ± 2.3 ^a^
PH	14.17 ± 2.18 ^b^	1630 ± 310 ^c^	11.5 ± 3.7 ^bc^	11.60 ± 1.68 ^b^	79.4 ± 8.6 ^bc^
SH	0.75 ± 0.06 ^a^	160 ± 10 ^a^	21.4 ± 3.0 ^d^	0.55 ± 0.07 ^a^	72.6 ± 4.1 ^b^
Wort	0.03 ± 0.00 ^a^	2.0 ± 1.0 ^a^	5.4 ± 1.6 ^ab^	0.03 ± 0.00 ^a^	84.5 ± 8.9 ^bc^
Beer	0.02 ± 0.00 ^a^	0.3 ± 0.1 ^a^	1.7 ± 0.4 ^a^	0.02 ± 0.01 ^a^	91.3 ± 2.7 ^c^

^a,b,c,d,e^ Different superscripts indicate significant differences among different lipid extracts within the same lipid class (*p* < 0.05) when means are compared using a Tukey’s HSD multiple comparison test. Abbreviations: BSG = brewer’s spent grain; MG = malted grain; PH = pelleted hops; SH = spent hops.

**Table 2:** The fatty acid profile and volatile compounds detected in the total polar lipid (TPL) extracts of each sample are expressed as a percentage of the total volatile components detected by GC-MS (mean ± SD, *n* = 3).

Fatty Acids		Malt Grain	Spent Grain	Pelleted Hops	Spent Hops	Wort	Beer
8:0	Caprylic acid	ND	ND	0.04 ± 0.01 ^b^	0.02 ± 0.01 ^ab^	0.01 ± 0.00 ^a^	ND
10:0	Capric acid	ND	ND	0.06 ± 0.00 ^b^	ND	0.01 ± 0.00 ^a^	0.02 ± 0.01 ^a^
12:0	Lauric acid	0.17 ± 0.02 ^d^	ND	0.02 ± 0.00 ^a^	0.03 ± 0.01 ^a^	0.09 ± 0.01 ^c^	0.07 ± 0.00 ^b^
12:1	*cis*-Lauroleic acid	ND	ND	0.05 ± 0.01	ND	ND	ND
14:0	Myristic acid	0.62 ± 0.06 ^b^	0.69 ± 0.06 ^b^	0.33 ± 0.05 ^a^	1.04 ± 0.11 ^c^	1.58 ± 0.04 ^d^	1.55 ± 0.09 ^d^
14:1	*cis*-Myristoleic acid	ND	0.41 ± 0.11	ND	ND	ND	ND
15:0	Pentadecylic acid	0.31 ± 0.04 ^a^	0.22 ± 0.07 ^a^	0.49 ± 0.14 ^b^	0.25 ± 0.01 ^a^	0.17 ± 0.02 ^a^	0.16 ± 0.02 ^a^
16:0	Palmitic acid	19.83 ± 0.93 ^a^	27.86 ± 0.80 ^b^	22.05 ± 1.23 ^a^	30.38 ± 1.0 ^c^	31.80 ± 0.60 ^c^	32.34 ± 0.68 ^c^
16:1	*cis*-Palmitoleic acid	0.39 ± 0.04 ^a^	0.30 ± 0.12 ^a^	2.35 ± 0.26 ^c^	1.15 ± 0.15 ^b^	0.60 ± 0.02 ^a^	0.33 ± 0.21 ^a^
17:0	Margaric acid	0.18 ± 0.02 ^a^	ND	1.24 ± 0.03 ^d^	0.46 ± 0.03 ^c^	0.24 ± 0.02 ^b^	0.23 ± 0.01 ^ab^
17:1	*cis*-Heptadecenoic acid	0.09 ± 0.01 ^a^	ND	0.75 ± 0.04 ^d^	0.36 ± 0.03 ^c^	0.20 ± 0.01 ^b^	0.20 ± 0.03 ^b^
18:0	Stearic acid	2.63 ± 0.62 ^ab^	2.23 ± 0.25 ^a^	2.85 ± 0.08 ^abc^	3.85 ± 0.18 ^d^	3.61 ± 0.09 ^cd^	3.32 ± 0.10 ^bcd^
18:1 c9	*cis*-Oleic acid	9.04 ± 0.19 ^d^	8.81 ± 0.44 ^d^	4.39 ± 0.11 ^a^	6.67 ± 0.26 ^d^	6.12 ± 0.24 ^bc^	5.62 ± 0.18 ^b^
18:1 t13	*trans*-Oleic acid	0.66 ± 0.02 ^a^	1.01 ± 0.09 ^ab^	1.27 ± 0.16 ^bc^	1.82 ± 0.24 ^d^	1.42 ± 0.09 ^c^	1.26 ± 0.14 ^bc^
18:2 c9, c12	Linoleic acid	56.67 ± 0.77 ^e^	51.83 ± 1.59 ^d^	25.46 ± 1.5 ^a^	40.68 ± 0.34 ^b^	44.78 ± 0.06 ^c^	43.48 ± 1.55 ^bc^
18:3 c6, c9, c12	γ-Linolenic acid	ND	ND	0.58 ± 0.02 ^b^	0.12 ± 0.01 ^a^	ND	ND
18:3 c9, c12, c15	α-Linolenic acid	6.80 ± 0.95 ^abc^	5.87 ± 0.54 ^ab^	23.42 ± 1.4 ^d^	8.83 ± 0.92 ^c^	7.72 ± 0.71 ^bc^	5.13 ± 0.26 ^a^
20:0	Arachidic acid	0.58 ± 0.10 ^ab^	0.78 ± 0.17 ^bc^	1.02 ± 0.04 ^c^	0.48 ± 0.07 ^a^	ND	ND
20:1 c13	Eicosenoic acid	ND	ND	0.25 ± 0.05 ^a^	0.54 ± 0.04 ^c^	0.39 ± 0.02 ^b^	ND
20:2 c11, c14	Eicosadienoic acid	ND	ND	1.00 ± 0.05 ^c^	0.49 ± 0.07 ^b^	0.27 ± 0.01 ^a^	0.31 ± 0.04 ^a^
20:4 c5, c8, c11, c14	Arachidonic acid	ND	ND	ND	ND	0.52 ± 0.06	4.93 ± 0.02
20:5 c5, c8, c11, c14, c17	Eicosapentaenoic acid	ND	ND	0.53 ± 0.01 ^b^	0.16 ± 0.03 ^a^	0.41 ± 0.10 ^b^	ND
22:0	Behenic acid	0.30 ± 0.04 ^a^	ND	1.32 ± 0.32 ^c^	0.72 ± 0.13 ^b^	0.41 ± 0.02 ^ab^	0.29 ± 0.07 ^a^
22:1	Erucic acid	0.32 ± 0.09 ^a^	ND	0.41 ± 0.06 ^a^	0.34 ± 0.09 ^a^	ND	ND
22:6 c4, c7, c10, c13, c16, c19	Docosahexaenoic acid	0.47 ± 0.09 ^a^	ND	1.46 ± 0.17 ^b^	0.43 ± 0.13 ^a^	ND	ND
Σ_SFA_		24.43 ± 0.70 ^a^	31.79 ± 0.94 ^b^	29.40 ± 1.32 ^b^	37.23 ± 0.91 ^c^	37.94 ± 0.60 ^c^	37.97 ± 0.54^c^
Σ_MUFA_		10.67 ± 0.14 ^c^	10.53 ± 0.41 ^c^	9.47 ± 0.48 ^b^	10.86 ± 0.37 ^c^	9.13 ± 0.21 ^b^	7.42 ± 0.45 ^a^
Σ_PUFA_		63.95 ± 1.53 ^c^	57.69 ± 1.09 ^b^	52.87 ± 2.66 ^a^	50.71 ± 0.67 ^a^	53.69 ± 0.60 ^a^	53.75 ± 1.76 ^a^
Volatiles							
Hexanedioic acid		ND	ND	ND	ND	0.28 ± 0.04	1.12 ± 0.21
Aromadendrene oxide		ND	ND	1.77 ± 0.39	0.11 ± 0.02	ND	ND
2,4-Di-*tert*-butylphenol		ND	ND	ND	ND	ND	0.12 ± 0.01
β-Caryophyllene		ND	ND	2.02 ± 0.37 ^b^	0.37 ± 0.05 ^a^	ND	0.07 ± 0.01 ^a^
2-Dodecanone		ND	ND	0.07 ± 0.05	0.04 ± 0.01	ND	ND
Cubenol		ND	ND	0.24 ± 0.18	ND	ND	ND
Tau-Cadinol		ND	ND	0.14 ± 0.08	ND	ND	ND
Tau-Muurolol		ND	ND	0.29 ± 0.02	ND	ND	0.08 ± 0.00
Σ_Volatiles_		ND	ND	8.90 ± 0.32 ^b^	0.95 ± 0.32 ^a^	0.28 ± 0.04	1.37 ± 0.22 ^a^

^a,b,c,d,e^ Mean values ± SD (*n* = 3), different letters in the same row indicate statistically significant differences between the lipid compositions when means are compared using Tukey’s HSD multiple comparison test (*p* ≤ 0.05). Abbreviations: c = *cis*; MUFA = monounsaturated fatty acids; ND: non-detectable; PUFA = polyunsaturated fatty acids; SFA = saturated fatty acids; t = *trans*.

**Table 3:** The in vitro biological activities of the total lipids (TL), total neutral lipids (TNL), and total polar lipids (TPL) of the beer and brewing by-products against platelet-activating factor (PAF)-induced human platelet aggregation, expressed as an IC_50_ in micrograms (µg) of the sample extract. The hPRP concentration was approximately 500,000 platelets µL^−1^. The final concentration of PAF in the cuvette was 2.6 × 10^−8^ M. All experimental analyses were carried out in triplicate (mean ± SD, *n* = 3).

Sample	TL	TNL	TPL
MG	495 ± 105 ^b^	298 ± 89 ^a^	191 ± 58 ^ab^
BSG	69 ± 33 ^a^	610 ± 136 ^b^	617 ± 184 ^c^
PH	453 ± 109 ^b^	1088 ± 172 ^c^	473 ± 280 ^c^
SH	519 ± 81 ^b^	924 ± 166 ^c^	436 ± 142 ^bc^
Wort	70 ± 29 ^a^	175 ± 61 ^a^	58 ± 11 ^a^
Beer	6.4 ± 4.5 ^a^	248 ± 66 ^a^	7.8 ± 3.9 ^a^

^a,b,c^ Different superscripts indicate significant differences among different lipid extracts within the same lipid class (*p* < 0.05), when means are compared using ANOVA and Tukey’s HSD multiple comparison test. Abbreviations: BSG = brewer’s spent grain; hPRP = human platelet-rich plasma; MG = malt grain PAF = platelet-activating factor; PH = pelleted hops; SH = spent hops; TL = total lipids; TNL = total neutral lipids; TPL = total polar lipids.

**Table 4:** The in vitro biological activities of the total lipids (TL), total neutral lipids (TNL), and total polar lipids (TPL) of the beer and brewing by-products against thrombin-induced human platelet aggregation. Results are expressed as an IC_50_ in micrograms (µg) of each lipid extract. The hPRP concentration was approximately 500,000 platelets µL^−1^. The final concentration of thrombin in the cuvette was 0.1–1.0 mU/mL. All experimental analyses were carried out in triplicate (mean ± SD, *n* = 3).

Sample	TL	TNL	TPL
MG	112 ± 21 ^b^	433 ± 77 ^b^	247 ± 39 ^b^
BSG	87 ± 10 ^b^	409 ± 30 ^b^	203 ± 49 ^b^
PH	221 ± 42 ^c^	478 ± 97 ^b^	207 ± 51 ^b^
SH	155 ± 56 ^bc^	572 ± 76 ^b^	396 ± 62 ^c^
Wort	10 ± 3.7 ^a^	165 ± 61 ^a^	24 ± 17 ^a^
Beer	2.4 ± 0.9 ^a^	206 ± 73 ^a^	4.3 ± 3.0 ^a^

^a,b,c^ Different superscripts indicate significant differences among different lipid extracts within the same lipid class (*p* < 0.05), when means are compared using ANOVA with Tukey’s HSD multiple comparison test. Abbreviations: BSG = brewer’s spent grain; hPRP = human platelet-rich plasma; PAF = platelet-activating factor; MG = malt grain; PH = pelleted hops; SH = spent hops; TL = total lipids; TNL = total neutral lipids; TPL = total polar lipids.
